# Triplon current generation in solids

**DOI:** 10.1038/s41467-021-25494-7

**Published:** 2021-08-31

**Authors:** Yao Chen, Masahiro Sato, Yifei Tang, Yuki Shiomi, Koichi Oyanagi, Takatsugu Masuda, Yusuke Nambu, Masaki Fujita, Eiji Saitoh

**Affiliations:** 1 Institute for Materials Research, Tohoku University, Sendai, Japan; 2 Department of Physics, Ibaraki University, Mito, Ibaraki Japan; 3 Department of Basic Science, The University of Tokyo, Tokyo, Japan; 4 Faculty of Science and Engineering, Iwate University, Morioka, Japan; 5 Institute of Solid State Physics, The University of Tokyo, Kashiwa, Chiba Japan; 6 FOREST, Japan Science and Technology Agency, Kawaguchi, Saitama Japan; 7 Organization for Advanced Studies, Tohoku University, Sendai, Japan; 8 Department of Applied Physics, The University of Tokyo, Tokyo, Japan; 9 Institute for AI and Beyond, The University of Tokyo, Tokyo, Japan; 10 Advanced Institute for Materials Research, Tohoku University, Sendai, Japan; 11 Advanced Science Research Center, Japan Atomic Energy Agency, Tokai, Japan

**Keywords:** Magnetic properties and materials, Spintronics

## Abstract

A triplon refers to a fictitious particle that carries angular momentum *S*=1 corresponding to the elementary excitation in a broad class of quantum dimerized spin systems. Such systems without magnetic order have long been studied as a testing ground for quantum properties of spins. Although triplons have been found to play a central role in thermal and magnetic properties in dimerized magnets with singlet correlation, a spin angular momentum flow carried by triplons, a triplon current, has not been detected yet. Here we report spin Seebeck effects induced by a triplon current: triplon spin Seebeck effect, using a spin-Peierls system CuGeO_3_. The result shows that the heating-driven triplon transport induces spin current whose sign is positive, opposite to the spin-wave cases in magnets. The triplon spin Seebeck effect persists far below the spin-Peierls transition temperature, being consistent with a theoretical calculation for triplon spin Seebeck effects.

## Abstract (web summary)

Triplons are elementary spin excitations characteristic of dimerized magnets, carrying a spin angular momentum one. Here, the authors report evidence for spin current of triplons via thermal transport measurements in a spin-dimer compound CuGeO_3_.

## Introduction

Spin Seebeck effects^1^ (SSEs) refer to the generation of a spin current, a flow of spin angular momentum of electrons, from a temperature gradient applied to a bilayer system comprising a magnet and heavy metal such as Pt. Spin current in the magnet propagates along the temperature gradient and reaches the heavy metal^2^, in which spin current can be detected as a voltage signal via the inverse spin-Hall effect (ISHE)^3,4^. The SSE has been observed in various insulating systems, in which parasitic thermal effects originating from itinerant electrons can be eliminated. Some types of spin carriers including magnons in magnets^5–8^, paramagnons^9^, antiferromagnetic magnons^7,8^, and spinons in a spin liquid^10,11^ have been demonstrated for different mechanisms of the SSE.

Among quantum spin systems without magnetic order, dimerized magnets spin systems occupy an important position, in which two neighboring spins are frozen as *S* = 0 singlets in the ground state. The elementary spin excitations are *S* = 1 triplet states, called a triplon. A triplon has been expected to carry spin angular momentum.

In this work, we report the observation of spin current in CuGeO_3_, carried by triplons in terms of longitudinal SSE measurements. A typical spin-dimer system is a spin-Peierls material CuGeO_3_, which contains one-dimensional spin-1/2 chains with antiferromagnetic exchange interaction for nearest-neighbor spins. In the spin chain, with lowering the temperature, neighboring spins dimerizes to form a spin-gapped phase; the transition is called a spin Peierls (SP) transition^12^. Below the SP transition temperature *T*_SP_, the chain distorts so that the distances between neighboring spins change alternately. The bond-alternating exchange interaction causes neighboring spins to dimerize to reduce the total energy, creating a gap in the spin excitation energy spectrum. CuGeO_3_ is the firstly discovered inorganic system that exhibits a SP transition^13^. High-quality single crystals of CuGeO_3_ are easier to obtain than organic SP materials and CuGeO_3_ has typically been used to study spin excitations in SP systems^14^.

## Results

### Sample structure and the concept of the study

**Fig. 1:**
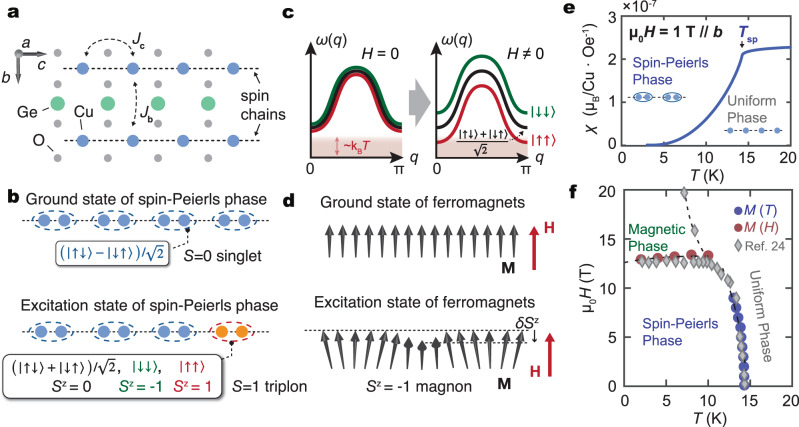
Spin chain and spin-Peierls (SP) phase of CuGeO_3_. **a** Crystalline structure of CuGeO_3_. The Cu spin chains are shown as dotted lines. Spin-1/2 spin chains along the *c*-axis in CuGeO_3_ are formed by staking CuO_2_ chains. *J*_*c*_ and *J*_*b*_ denote the exchange interactions along the *c* and *b*-axis, respectively. **b** When temperature (*T*) falls down to the SP transition temperature *T*_SP_, CuGeO_3_ undergoes a SP transition. After the transition, the Cu spins dimerize and the ground state becomes a spin singlet state ($(\left|\uparrow \downarrow \right\rangle -\left|\downarrow \uparrow \right\rangle )/\sqrt{2}$ with *S* = 0). Elementary excitation in the SP phase is a triplon with *S* = 1. **c** Schematic band structure of triplon in CuGeO_3_^19,21^. Three triplet states ($\left|\downarrow \downarrow \right\rangle$, $(\left|\uparrow \downarrow \right\rangle +\left|\downarrow \uparrow \right\rangle )/\sqrt{2}$ and $\left|\uparrow \uparrow \right\rangle$) are threefold degenerated at zero fields. By applying a magnetic field (*H*), the Zeeman effect lift the degeneracy. **d** The ground state and excitation state of a ferromagnet. Elementary excitation is a magnon with *S* = 1. The magnons reduce the magnetization (**M**) along the magnetic field (**H**) as *δS*^*z*^. **e ***T* dependence of magnetic susceptibility (*χ*) of CuGeO_3_ under an applied magnetic field of 1 T along the *b*-axis. The SP transition temperature *T*_SP_ is illustrated as the arrow in the figure. **f** Magnetic field - temperature (*H* - *T*) spin phase diagram for the CuGeO_3_ sample obtained from the *M*(*H*) and *χ*(*T*) measurement results. The diamond symbols represent data taken from a previous study^24^. The dotted curves are guide for the eyes.

Figure 1a shows the crystal structure of CuGeO_3_. One-dimensional Cu^2+^ spin (*S* = 1/2) chains are aligned along the *c*-axis, as illustrated as dotted lines. The nearest-neighbor exchange interaction along the *c*-axis is *J*_*c*_ ~ 120 K, much greater than the interchain exchange coupling^15^, *J*_*b*_ ~ 0.1*J*_*c*_, and *J*_*a*_ ~ −0.01*J*_*c*_ along the *b* and *a*-axis, respectively. CuGeO_3_ is thus considered as a quasi-one-dimensional spin system. As the temperature *T* decreases below the SP transition temperature *T*_SP_ ~ 14.5  K, the lattice of CuGeO_3_ is spontaneously distorted to form a bond alternating configuration^16^. The spin configuration in the ground state is shown schematically in Fig. 1b, where all spins are frozen as dimerized *S* = 0 singlets. The elementary spin excitation from the ground state is an *S* = 1 triplon^17^, also shown in Fig. 1b. The excitation gap of a triplon is estimated to be ~23 K from ESR^18^ and neutron scattering^15,19,20^ experiments.

Based on the previous neutron scattering results^19^ and theoretical calculations^21^, the dispersion relation of triplons along the *c*-axis is sketched in Fig. 1c. In the absence of a magnetic field, *μ*_0_*H* = 0, the triplon states are threefold degenerated: $\left|\uparrow \uparrow \right\rangle$, $(\left|\uparrow \downarrow \right\rangle +\left|\downarrow \uparrow \right\rangle )/\sqrt{2}$, and $\left|\downarrow \downarrow \right\rangle$ corresponding to states with different spin quantum numbers, *S*_*z*_ = 1, 0, and −1, respectively. When a magnetic field is applied, the degenerated triplet bands split into three due to the Zeeman effect, where the triplet state with the parallel spin direction to *H*, $\left|\uparrow \uparrow \right\rangle$, has the lowest energy^18–20^. The $\left|\uparrow \uparrow \right\rangle$ state thus exhibits the highest probability for thermal excitation, while $\left|\downarrow \downarrow \right\rangle$ the lowest. Driven by a thermal gradient, different occupancy among the three states can result in a flow of a net spin angular momentum in an external magnetic field: triplon SSE. One of the key features of the triplon SSE is that it should exhibit the opposite sign of ISHE signal to the magnon SSE in ferri- and ferro- magnets. This is because triplon spin currents are carried by excitation with the parallel spin direction to the external field; while magnons carry antiparallel spins to the external field, as shown in Fig. 1d. In these two systems, the interfacial accumulated spins in the magnets are injected into the metal (Pt) through the interfacial exchange interaction. Since the exchange interaction conserves the spin angular momentum, the spin current in the metal has the same polarization direction as that in the magnets. As a result, the sign of ISHE in the triplon SSE is opposite to that in the magnon SSE. Our theoretical calculation based on the tunnel spin current (see Supplementary Note G3 for details) suggests that the opposite sign of triplon SSE to magnon SSE is a universal property for gapped triplet-spin systems.

### Material characterization and measurement setup

First, we measured the temperature dependence of magnetization, *M*(*T*), for the CuGeO_3_ sample. Figure 1e shows the magnetic susceptibility *χ*(*T*) = *M*(*T*)/*H* measured at *μ*_0_*H* = 1 T ∥*b*-axis. When *T* is decreased from 20 K, *χ* exhibits a sudden drop at the SP transition temperature *T*_SP_ ~ 14.5 K and rapidly decreases towards zero. This indicates that Cu spins along the spin chain (∥*c*-axis) form dimers and a spin gap develops below *T*_SP_. By measuring *χ*(*T*) at different values of *H* (see Supplementary Fig. 1 for details), we obtain the *H*–*T* spin-phase diagram of the CuGeO_3_ sample in Fig. 1f. In the phase diagram, for large *H* in the SP phase, CuGeO_3_ undergoes another phase transition into a magnetic phase at a transition field, *H*_m_, where magnetization is recovered by forming a spin soliton lattice in the chain^22^. The transition can be confirmed as a sudden jump of magnetization as a function of fields^23^ (also see Supplementary Fig. 1). The obtained phase diagram is in good agreement with a previous study^24^.

**Fig. 2:**
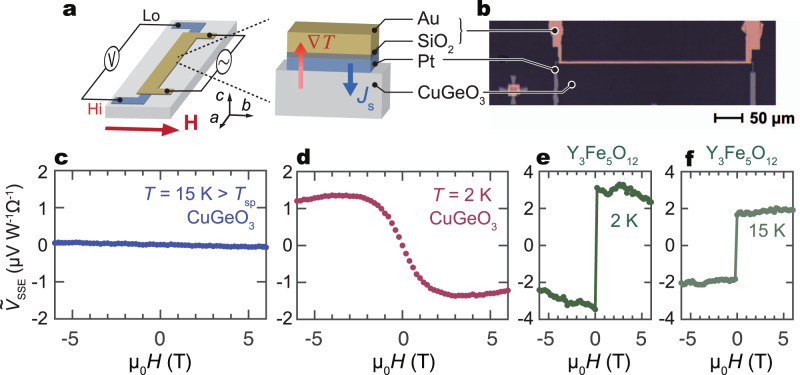
SSE measurement results for CuGeO_3_/Pt. **a** Experimental setup for the SSE measurement. Temperature gradient (∇*T*) is applied by using an Au heater electrically insulated from the Pt layer by a SiO_2_ film. An a.c. current is applied to the Au heater and SSE voltage in the Pt is measured with a lock-in amplifier as the second harmonic voltage *V*_2*f*_. **b** An optical micrograph for a on-chip SSE device made on the top of CuGeO_3_. **c**, **d** The magnetic field (*H*) dependence of ${\tilde{V}}_{{{{{{{{\rm{SSE}}}}}}}}}$ (the SSE signal normalized to the heating power and the detector resistance) for the CuGeO_3_/Pt sample at 15 and 2 K, respectively. **e**, **f **${\tilde{V}}_{{{{{{{{\rm{SSE}}}}}}}}}(H)$ measured for Y_3_Fe_5_O_12_/Pt at 2 and 15 K.

The experimental setup for the SSE measurement and the sample used in the present study are shown in Fig. 2a, b. A trilayer structure comprising a Pt wire for ISHE, an insulating SiO_2_ layer, and an Au wire as a heater was fabricated on the top of the (001) plane of a CuGeO_3_ single crystal. The spin Seebeck voltage signal *V*_SSE_ in the Pt wire is measured by a lock-in method, where an a.c. current is applied to the Au layer under an in-plane magnetic field, *H*, applied perpendicular to the Pt wire. We normalize *V*_SSE_ by the heater power and the detector resistance as ${\tilde{V}}_{{{{{{{{\rm{SSE}}}}}}}}}={V}_{{{{{{{{\rm{SSE}}}}}}}}}/({P}_{{{{{{{{\rm{Au}}}}}}}}}\cdot {R}_{{{{{{{{\rm{Pt}}}}}}}}})$.

### Observation of the triplon SSE

The magnetic field dependence of ${\tilde{V}}_{{{{{{{{\rm{SSE}}}}}}}}}$ at some selected temperatures is shown in Fig. 2c, d. At 15 K, just above *T*_SP_ ~ 14.5 K, no *H*-dependent signal is recognized. Most notably, a clear voltage signal appears when *T* is decreased down to 2 K. The signal is an odd function of *H*, reflecting the symmetry of the ISHE^25^. The sign of the ${\tilde{V}}_{{{{{{{{\rm{SSE}}}}}}}}}$ signal is opposite to that of the magnon-mediated SSE^26,27^, which is confirmed in a similar setup for a ferrimagnetic insulator Y_3_Fe_5_O_12_/Pt sample in Fig. 2e, f. The result indicates that spin current carriers in these two systems have different spin polarization directions with respect to *H*, consistent with the triplon-current senario. Note that the sign of the SSE signals in spin nematic LiCuVO_4_/Pt (ref. ^28^) is also opposite to the present CuGeO_3_/Pt case. We also note that, unlike the magnon-mediated SSE in ferro/ferrimagnets, the triplon-mediated SSE does not scale with the *M*(*H*) curve. For the magnon-mediated SSE^27^, *M*(*H*) represents the increase in the spin-current polarization. In contrast, the magnetization increase in the SP phase means larger density of broken dimers and the scattering probability between triplons and broken dimers becomes larger. The unusual *H*-dependence of the triplon SSE originates from the different properties between ferro/ferrimagnets and SP phases, and it is one of the characteristics of triplon SSE. (see Supplementary Fig. 2 for details).

**Fig. 3:**
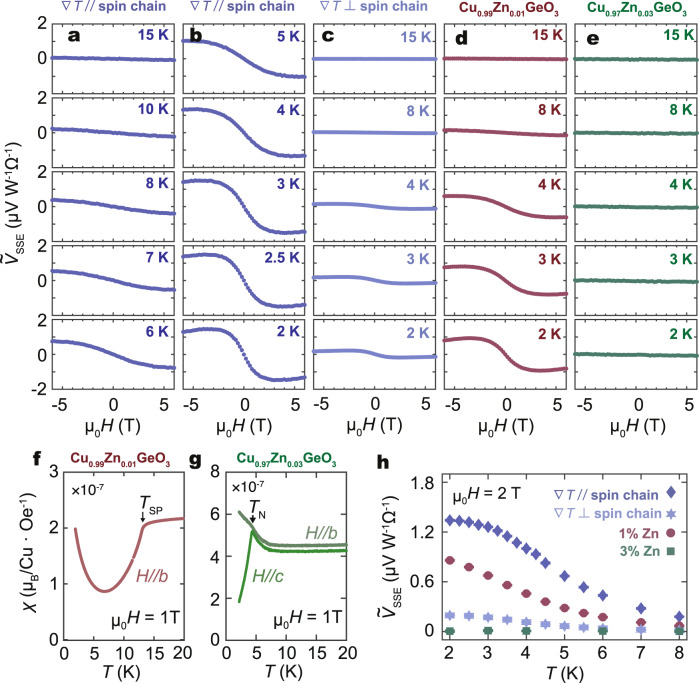
Temperature dependence of SSE signal. **a**, **b** Magnetic field (*H*) dependence of ${\tilde{V}}_{{{{{{{{\rm{SSE}}}}}}}}}$ (the SSE signal normalized to the heating power and the detector resistance) at selected temperatures (*T*) for CuGeO_3_/Pt, with temperature gradient (∇*T*) applied along the spin chain. **c** *H* dependence of ${\tilde{V}}_{{{{{{{{\rm{SSE}}}}}}}}}$ for CuGeO_3_/Pt, with ∇*T* applied perpendicular to the spin chain. **d**, **e** *H* dependence of ${\tilde{V}}_{{{{{{{{\rm{SSE}}}}}}}}}$ at selected temperatures for Cu_0.99_Zn_0.01_GeO_3_/Pt and Cu_0.97_Zn_0.03_GeO_3_/Pt, respectively. In **a**–**e**, the SSE signal is normalized by the heating power and the detector resistance. **f**, **g ***T* dependence of magnetic susceptibility for Cu_0.99_Zn_0.01_GeO_3_ and Cu_0.97_Zn_0.03_GeO_3_, respectively. **h ***T* dependence of SSE signal in different samples at *μ*_0_*H* = 2 T. Error bars represent standard deviation.

As shown in Fig. 3a, b, the temperature dependence of ${\tilde{V}}_{{{{{{{{\rm{SSE}}}}}}}}}$ shows that the signal magnitude increases monotonically as *T* decreases from *T*_SP_ to 3 K. When *T* < 3 K, the SSE voltage remains almost unchanged down to 2 K. At higher temperatures, the dimerized ground state is weakened because the thermally excited phonon disturbs the spontaneous displacement of the Cu^2+^ ion. In other words, the singlet ground state and triplon excitation is no longer a good picture for the low-energy excitation, and the triplon lifetime shrinks toward higher temperatures and completely disappears at *T*_SP_. This effect has been observed as an increase in the peak width of the triplon excitation in neutron scattering experiments^29^. We note that, by phenomenologically incorporating the triplon-phonon scattering term in a calculation, the *T* dependence of ${\tilde{V}}_{{{{{{{{\rm{SSE}}}}}}}}}$ at moderate temperatures can be explained qualitatively (see Supplementary Note G4 for details).

### Influence of crystal orientation and Zn-doping on the triplon SSE

Let us turn to the *H* dependence of ${\tilde{V}}_{{{{{{{{\rm{SSE}}}}}}}}}$ in CuGeO_3_/Pt. In the entire temperature range, as *H* increases from zero, ${\tilde{V}}_{{{{{{{{\rm{SSE}}}}}}}}}$ increases first and takes its maximum at *μ*_0_*H* ~ 2 T. As *H* further increases, ${\tilde{V}}_{{{{{{{{\rm{SSE}}}}}}}}}$ is then suppressed. As shown theoretically in the following, triplon scattering by inevitable impurities and defects in the nominally pure CuGeO_3_ sample can explain the observed *H* dependence of ${\tilde{V}}_{{{{{{{{\rm{SSE}}}}}}}}}$.

To show that the observed voltage is related to the triplon in the SP phase, we performed another control experiment with ∇*T* applied perpendicular to the spin chain configuration. Figure 3c shows ${\tilde{V}}_{{{{{{{{\rm{SSE}}}}}}}}}(H)$ for ∇*T*⊥*c* in a reference sample prepared with the same procedure as ∇*T*∥*c*. The observed SSE signal in ∇*T*⊥*c* is only 1/7 in magnitude as compared to the ∇*T*∥*c* configuration, consistent with the highly anisotropic transport of triplons. This result corroborates that the origin of the observed signal is the 1-D triplon transport. Moreover, additional reference experiments are performed for two nonmagnetic Zn-doped CuGeO_3_ samples, in which the triplon spin current should be suppressed by Zn-induced scattering. Figure 3d, e show the ${\tilde{V}}_{{{{{{{{\rm{SSE}}}}}}}}}(H)$ for Cu_0.99_Zn_0.01_GeO_3_/Pt and Cu_0.97_Zn_0.03_GeO_3_/Pt in the same temperature range as the CuGeO_3_/Pt measurements. Clearly, ${\tilde{V}}_{{{{{{{{\rm{SSE}}}}}}}}}$ is suppressed in the 1% Zn-doped sample and disappeared in the 3% Zn-doped sample.

Figure 3f, g shows *χ* for Cu_0.99_Zn_0.01_GeO_3_ and Cu_0.97_Zn_0.03_GeO_3_, respectively. For Cu_0.99_Zn_0.01_GeO_3_, the SP transition is observed at 13.2 K. The decrease of *T*_SP_ in doped CuGeO_3_ agrees with the result from the previous reports^30^. On the other hand, the SP transition is not visible in *χ*(*T*) in Cu_0.97_Zn_0.03_GeO_3_. Instead, a clear cusp is observed in *χ*_*c*_(*T*) (with *H*∥*c*-axis) at *T*_N_ ~ 4.3 K, indicating phase transition to an antiferromagnetic phase. The *T*_N_ is consistent with the previously reported value for Zn-3% doped CuGeO_3_ (ref. ^30^). The doping effect implies that non-magnetic Zn breaks the Cu-spin chains and introduces unpaired Cu free spins, called solitons, into the spin chain. Solitons disturb the dimerization, and local antiferromagnetic correlation may lead to long-range antiferromagnetic order^31^ at low temperatures in Cu_0.97_Zn_0.03_GeO_3_. As shown in Fig. 3h, ${\tilde{V}}_{{{{{{{{\rm{SSE}}}}}}}}}$ is significantly suppressed in the ∇*T*⊥ spin-chain configuration and the Zn-doped CuGeO_3_ samples in all *T* range. In the doped samples, the spin-excitation gap is still present in the SP phase, but their transport may be blocked by Zn. The result suggests that the triplon spin current is strongly influenced by Zn-induced scattering. Curie-Weiss fitting of *χ*(*T*) at low *T* (<3 K)^13,32^ (see Supplementary Note F for details) reveals that the density of free *S* = 1/2 spins is estimated to be 0.02% in the nominally pure CuGeO_3_ sample, so the effect of triplon scattering by impurities cannot be ignored. Therefore, we attempted to formulate the triplon spin current in the manner of Boltzmann equation, which is capable of incorporating scattering effects.

### Theoretical model for the triplon SSE

**Fig. 4:**
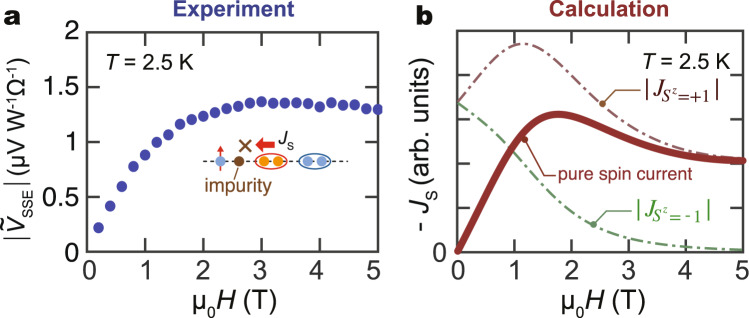
Magnetic field dependence of SSE voltage. **a** Magnetic field (*H*) dependence of the magnitude of $| {\tilde{V}}_{{{{{{{{\rm{SSE}}}}}}}}}|$ (absolute value of the SSE signal normalized to the heating power and the detector resistance) at 2.5 K. **b** Calculation results of the *H* dependence of the triplon spin current (see text and Supplementary Note G2 for details).

Figure 4 compares the *H* dependence of the observed SSE voltage signal with theoretical results. As we already discussed, $| {\tilde{V}}_{{{{{{{{\rm{SSE}}}}}}}}}|$ is expected to monotonically grow with increasing *H* because the triplon density of *S*_*z*_ = 1, i.e., the dominant carrier of spin current, increases with lowering the spin gap up to the critical field *H*_m_. However, the observed broad peak (see Fig. 4a) and the Zn-doped results shown in Fig. 3 strongly suggest the importance of triplon scattering processes. To theoretically analyze triplon currents in CuGeO_3_, we here apply the Boltzmann equation^33^. The spin current *J*_*s*_ computed by the Boltzmann equation (See Supplementary Note G2) is given by

1 $${J}_{s}=\mathop{\sum}\limits_{{S}_{z}}\int \frac{k}{2\pi }\hslash {S}_{z}{({v}_{{S}_{z}}(k))}^{2}{\tau }_{k}{k}_{{{{{{{{\rm{B}}}}}}}}}{{\Delta }}T\frac{{\epsilon }_{{S}_{z}}(k)}{{({k}_{{{{{{{{\rm{B}}}}}}}}}T)}^{2}}\frac{\exp ({\epsilon }_{{S}_{z}}(k)/{k}_{{{{{{{{\rm{B}}}}}}}}}T)}{{(\exp \left[{\epsilon }_{{S}_{z}}(k)/{k}_{{{{{{{{\rm{B}}}}}}}}}T\right]-1)}^{2}},$$

where Δ*T* is the temperature difference along the spin chain direction. ${v}_{{S}_{z}}(k)$, *τ*_*k*_ and ${\epsilon }_{{S}_{z}}(k)$ are the group velocity, relaxation time, and energy dispersion for the triplons^17^, respectively. We assume that the triplon can be viewed as a bosonic particle and the final part of Eq. (1) stems from the Bose distribution function. The *S*_*z*_ = 0 branch of the triplon does not contribute to the spin current. At *H* = 0, the distribution functions of *S*_*z*_ = ±1 branches are identical, which cancel out the total spin current. By varying *T* and *H*, the thermal excitation and the Zeeman effect alter the triplon distributions. Assuming a constant *τ*_*k*_, the population difference between *S*_*z*_ = ±1 increases monotonically at a fixed *T*(<*T*_SP_) due to the Zeeman effect, resulting in an almost *H*-linear increase in *J*_*s*_. The *H*-linear behavior can also be reproduced by using a tunnel spin-current formula^10,28,34,35^, which is another microscopic theory for thermal spin current (see Supplementary Note G3).

As shown in Fig. 4a, $| {\tilde{V}}_{{{{{{{{\rm{SSE}}}}}}}}}(H)|$ takes a maximum at around 3 T. To explain the non-monotonic behavior of ${\tilde{V}}_{{{{{{{{\rm{SSE}}}}}}}}}$, we considered two mechanisms of triplon scattering processes. One is the scattering by nonmagnetic impurities, where the scattering probability 1/*τ*_*k*,non-mag_ is constant with respect to *H*. Another source of scattering is free spins of broken dimers and unpaired Cu^2+^ (*S* = 1/2). The density of scatterers and scattering potential are assumed to depend on *H* as a Brillouin function. The total scattering probability is then the sum of the two scattering mechanisms, 1/*τ*_*k*,total_ = 1/*τ*_*k*,non-mag_ + 1/*τ*_*k*,mag_. Assuming that the scattering effect by magnetic impurities is dominant, we can reproduce the experimentally obtained *H* dependence of ${\tilde{V}}_{{{{{{{{\rm{SSE}}}}}}}}}$ shown in Fig. 4b. In the low *H* regime, where the spin of magnetic impurities is not aligned and the scattering effect is weak, the Zeeman effect dominates the generation and transport of the triplon current. With increasing *H*, the triplon mode of *S*_*z*_ = +1 (*S*_*z*_ = −1) shifts downward (upward) due to the Zeeman effect, and the imbalance between *S*_*z*_ = ±1 triplons becomes larger, resulting in an increase in the total triplon current. As *H* is further increased, the impurities are magnetized and magnetic scattering dominates the triplon transport, resulting in a broad peak as shown in Fig. 4b. The result agrees with the experimentally obtained data shown in Fig. 4a at a semi-quantitative level.

## Discussion

Finally, we here examine other possible origins for the observed thermoelectric voltage. One candidate could be the paramagnetic SSE due to free Cu spins. Several papers have addressed the spin current transport in paramagnets, including Gd_3_Ga_5_O_12_ (refs. ^9,36^), DyScO_3_ (ref. ^9^), and La_2_NiMnO_6_ (ref. ^37^). Paramagnon is believed to be responsible for spin transport in paramagnets as a result of short-range magnetic correlation or long-range dipole interactions. The spin diffusion length of Gd_3_Ga_5_O_12_ is estimated to be about 1.8 μm at 5 K^36^. However, in our CuGeO_3_ sample with the free spin density of 0.02%, the average distance between free spins is estimated to be around 0.29 nm/0.02% ~ 1.5 μm (the distance between neighboring Cu^2+^ ions along the *c*-axis is 0.29 nm^38^). This means that the paramagnetic spins are too dilute for spin correlation and spin current transport. Furthermore, the suppression of *V*_SSE_ in the Zn-doped samples, which exhibit larger paramagnetic moments than CuGeO_3_, also rules out the possibility of paramagnetic SSE.

The normal and anomalous Nernst effects may also contribute to an electric voltage under a temperature gradient, but these effects contradict with the suppression of the signal in the high-temperature spin-liquid phase; the vanishing signals in the ∇*T*⊥*c* configuration and the Zn-doped samples (see Fig. 3a–e). Furthermore, the normal Nernst effect should be linear with respect to the applied magnetic field and can not explain the observed magnetic field dependence of *V*_SSE_ shown in Fig. 3a, b.

Note that the voltage observed at 15 K in the CuGeO_3_/Pt shows almost no *H*-dependence, as shown in Fig. 2c. Above *T*_SP_, the elementary excitation of spin chains are gapless spinons^21^. A spinon spin current, a spin current carried by spinons, was found in Sr_2_CuO_3_ (ref. ^10^). The spinon spin current may contribute to a small *H*-linear voltage signal above *T*_SP_ (see Supplementary Note C for details). In the spin-Peierls phase, the gapless spinon is replaced by the gapped triplon^21^.

In summary, we observed the triplon spin-Seebeck effect in CuGeO_3_/Pt. Due to the triplon excitation in the system, the sign of the observed SSE is opposite to that of the conventional magnon SSE. The triplon SSE signal persists down to 2 K in the spin-Peierls phase and the magnetic field dependence of the triplon SSE is consistent with microscopic calculation. Our result shows that the spin-Seebeck effect also acts as a probe for spin excitations in gapped spin systems, and can also be applied to other materials with exotic spin excitation, such as a spin ladder system SrCu_2_O_3_ (ref. ^39^) and a spin dimer system SrCu_2_(BO_3_)_2_ (ref. ^40^).

## Methods

### Sample fabrication

CuGeO_3_ single crystals were grown by a traveling solvent floating zone (TSFZ) method. Samples were cut into size of ~7 mm × 3 mm for further measurements. 40nm-thick ferrimagnetic insulator Y_3_Fe_5_O_12_ single crystals were grown on Gd_3_Ga_5_O_12_(111) substrates by magnetron sputtering. Prior to the deposition, Gd_3_Ga_5_O_12_ substrates were annealed in the air at 825 ^∘^C for 30 min in a face-to-face configuration. To crystalize the as-grown amorphous Y_3_Fe_5_O_12_, the samples were post-annealed in the air at 825 ^∘^C for 200 s. For SSE measurements, on-chip devices with Pt detector and SiO_2_/Au heater were fabricated on top of samples (both pure/doped CuGeO_3_ and Y_3_Fe_5_O_12_) by electron-beam lithography, magnetron sputtering and lift-off technique (see Supplementary Note A).

### SSE measurement

We adapted the on-chip heating method for SSE, as illustrated in Fig. 1b. The resistance between the Au and Pt layers is much greater than MΩ at room temperature. A longitudinal temperature gradient was generated by applying an a.c. current with the frequency of *f* = 13 Hz to the Au heater. Since the SSE is driven by Joule heating, the frequency of the SSE signal on the Pt wire is 2*f*. The SSE signal was detected via a lock-in method.

### Magnetization measurement

The magnetization of CuGeO_3_ was measured in a Quantum Design Physical Properties Measurement System (PPMS) with a vibrating sample magnetometer (VSM) option.

## Supplementary information

Supplementary information

Peer Review File

## Data Availability

The data that support the findings of this study are available from the corresponding authors upon reasonable request. Source data are provided with this paper.

## Source data

Source Data

## Code availability

The codes used in theoretical simulations and calculations are available from the corresponding authors upon reasonable request.
