# Supplementary material for: Triplon current generation in solids
Source: Nat Commun. 2021 Aug 31;12:5199. doi: 10.1038/s41467-021-25494-7 (PMC8408157; doi:10.1038/s41467-021-25494-7)
Supplement: Supplementary file 1 — Supplementary information [file 41467_2021_25494_MOESM1_ESM.pdf]

Supplementary information for

Triplon current generation in solids

Yao Chen, Masahiro Sato, Yifei Tang, Yuki Shiomi, Koichi Oyanagi, Takatsugu Masuda, Yusuke Nambu, Masaki Fujita, & Eiji Saitoh

Supplementary Note A. Experimental details of spin-Seebeck effect measurements

Single crystalline $CuGeO_3$ prepared by a floating zone method[1] was an elliptical cylinder with the height of ~ 3 cm (along the a-axis), the long axis of 7 mm (along the c-axis) and a short axis of 3 mm (along the b-axis). Crystal orientations are determined by using a Laue camara. Firstly, a 340 μm long, 1.5 μm wide and 5 nm thick Pt wire was fabricated on $CuGeO_3$ by an e-beam lithography and lift-off process. Subsequently, a SiO_2/Au wire with the length of 360 μm and the width of 7.5 μm was fabricated on the top of the Pt wire. The thickness of the SiO_2 and Au are 8 nm and 80 nm, respectively. Au and Pt layers are insulated by a SiO_2 layer.

In the SSE measurement, a sinusoidal current (f = 13 Hz) was applied to the Au layer with a current source (Keithley 6221, Tektronix, Inc.). The voltage signal showing up along the Pt wire was recorded by a lock-in amplifier (NF 5640, NF Corporation). All SSE measurements were performed in a PPMS (Physical Property Measurement System, Quantum Design, Inc.).

Supplementary Note B. Estimation of T_{SP} and H_m of the $CuGeO_3$ sample

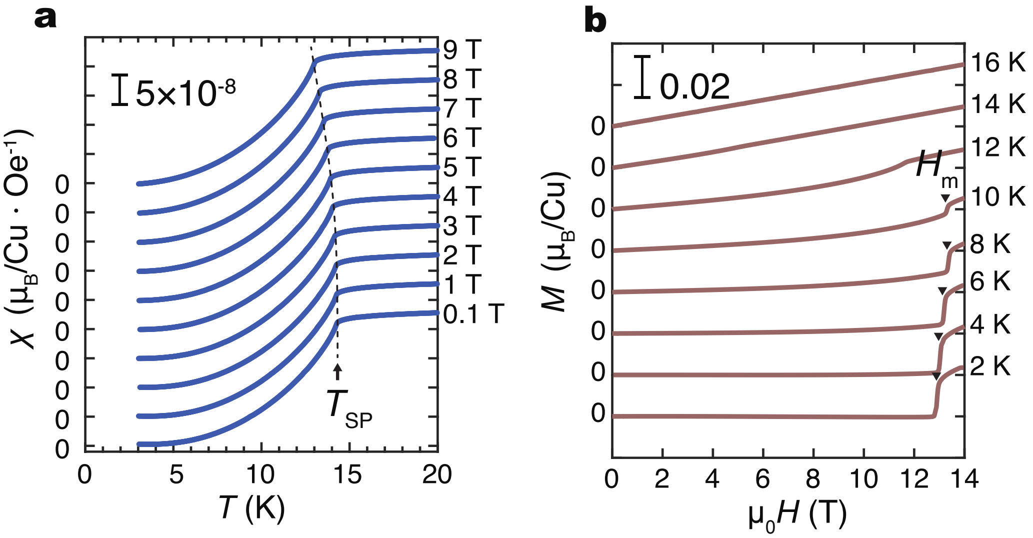

Supplementary Fig. 1. **Temperature (T) and magnetic field (H) dependence of the magnetization (M) of $CuGeO_3$. a,** T dependence of magnetic susceptibility ($\chi = M/H$) at different H values. **b,** H dependence of M at different T values. Data are obtained with H applied along the b-axis.

Magnetization (M) measurements were carried out using the VSM (vibrating sample magnetometer) option of PPMS. The $\chi(T) = M(T)/H$ results obtained under various H values are shown in Supplementary Fig. 1a. Based on the results, we determined the spin-Peierls (SP) transition temperature T_{SP} at each H. The transition field (H_{m}) from the SP phase to the magnetic phase at each T was determined by measuring $M(H)$. A steep increase in $M(H)$ was detected at μ_0H ~ 13 T for $T < T_{\mathrm{sp}}$, as shown in Supplementary Fig. 1b. H_{m} is defined as the field where $dM(H)/dH$ is maximized.

Supplementary Note C. Comparison between magnetization and SSE signal

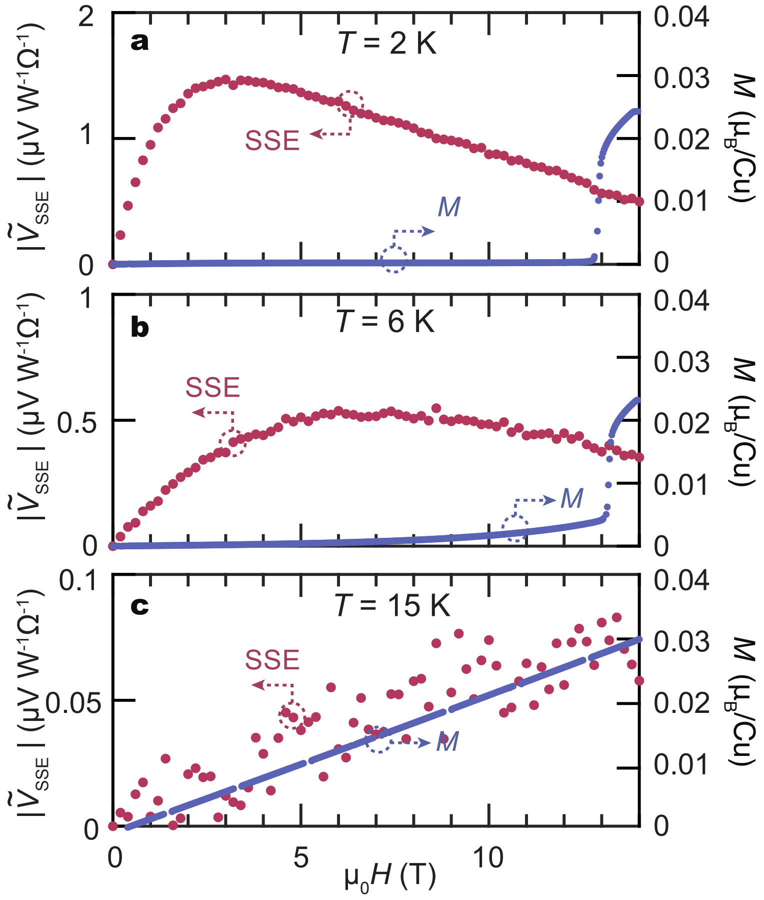

Supplementary Fig.2. **Comparison between magnetization M and the SSE signal $|V_{\mathrm{SSE}}|$. a-c,** at 2 K, 6 K and 15 K, respectively.

A comparison between $M(H)$ and SSE at different temperatures are shown in Supplementary Fig. 2. Unlike the case of the magnon SSE in most ferro/ferri-magnets, $V_{\mathrm{SSE}}(H)$ in $CuGeO_3$ does not scale with $M(H)$. The magnetization of $CuGeO_3$ at $T < T_{\mathrm{SP}}$ mainly consists of impurity spins and unpaired spins on thermally broken dimers. As shown in Supplementary Fig. 2a and 2b,

the SSE signal does not scale with M-H at $T = 2$ K and 6 K. The M-H in the low field range reflects the paramagnetic term from free (impurity) spins, and these free spins do not play a role in $V_{\mathrm{SSE}}(H)$ because they are well decoupled. At $T = 6$ K, the magnetic component from broken dimers overwhelms the paramagnetic component from impurity spins. Since the broken dimers are also little important for spin-current carriers in SSE, V_{SSE} and M should show totally different magnetic field dependence.

For $T = 15\ \mathrm{K} > T_{\mathrm{SP}}$, the system is theoretically predicted to be a type of paramagnets: a Tomonaga-Luttinger (TL) spin liquid, where the equilibrium state is still a zero magnetizated state in the absence of fields and the spin excitation is a gapless spinon instead of triplon. For higher temperatures ($k_{\mathrm{B}}T > J$), the system experience a crossover from the TL spin liquid to a paramagnetic state. We also expect a spinon SSE[2] in the intermediate temperature range ($T_{\mathrm{SP}} < T \ll J/k_{\mathrm{B}}$). From the paper by Hirobe, et al (ref. [2]), the spinon SSE signal in TL spin liquids was found to be much smaller than the ferromagnetic SSE, linear with respect to the magnetic field. The SSE signal measured at $15\ \mathrm{K} > T_{\mathrm{SP}}$ satisfies all these properties, and thus may be an indication of the spinon SSE.

Also, the anomaly in the SSE is hardly observed at H_{m}. This is because the net spin current above H_{m} is still determined by low-energy $S = +1$ triplons and high-energy $S = -1$ triplons, similar to the case of $H < H_{\mathrm{m}}$ (see Supplementary Fig. 3 which illustrates the triplon bands above H_{m}). As shown in Supplementary Fig. 3, for $H > H_{\mathrm{m}}$, the excitation energy of the $S = +1$ triplon is lower than the energy of the ground state, and the $S = +1$ triplon condenses. This is

called the triplon BEC (ref.[3]). The condensed triplon shows static magnetization but does not carry spin current.

The comparison between SSEs in an antiferromagnetic insulator and the SP magnet $CuGeO_3$ would be useful to deeply understand the feature of the triplon SSE. In the case of antiferromagnet/Pt system, the SSE signal changes abruptly near the spin-flop transition[4,5]. At the spin-flop transition, both the ground state and the magnon-band structure drastically change and a net magnetization suddenly appears because the spin-flop transition is of a first-order type. Due to this significant change of the magnon band, the SSE signal largely changes at the spin-flop transition. On the other hand, although the triplon BEC occurs for $H > H_{\rm m}$ of $CuGeO_3$, the band structure of the triplon excitation is very similar to that for $H < H_{\rm m}$. Therefore, there was no significant change in the triplon SSE signal around $H_{\rm m}$.

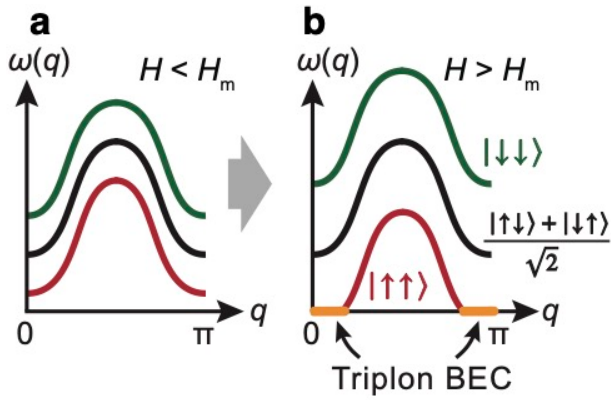

Supplementary Fig.3. **A schematic illustration of triplon BEC. a,** triplon band structure with magnetic field (H) less than the transition field (H_{m}) from the SP phase to the magnetic phase. **b,** triplon band structure with $H > H_{\mathrm{m}}$. Green, black and red curves represent three triplet states $|\downarrow\downarrow\rangle$, $\left(|\uparrow\downarrow\rangle + |\downarrow\uparrow\rangle\right)/\sqrt{2}$, and $|\uparrow\uparrow\rangle$ respectively.

Supplementary Note D. Angular and heater-power dependence of the spin-Seebeck signal

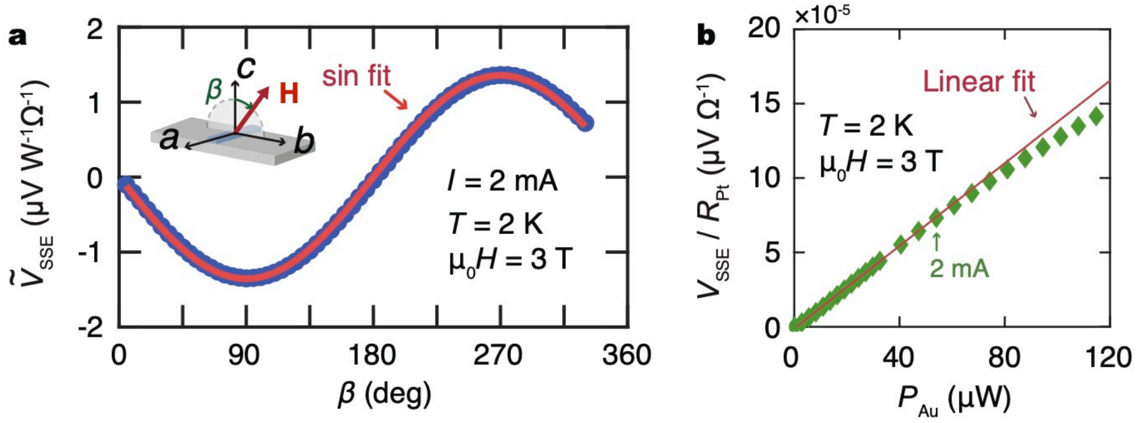

Supplementary Fig.4. **Angular and heating power dependence of the SSE signal. a,** Angular dependence of $\tilde{V}_{\rm SSE}$ at $\mu_0 H = 3$ T and $T = 2$ K, with a heating current (I) of 2 mA. The red curve indicates a sinusoidal fit. **b,** Heater-power dependence of $\tilde{V}_{\rm SSE}$ at $\mu_0 H = 3$ T and $T = 2$ K. The red line is a linear fit to the data for the heater currents ranging from 0 to 2 mA. Error bars represent 95% confidence interval of the sinusoidal fitted amplitude.

Supplementary Fig. 4a shows $\tilde{V}_{\rm SSE}(\beta)$ in $CuGeO_3$/Pt at $T = 2$ K and $\mu_0 H = 3$ T. Here, H is always perpendicular to the Pt wire and the out-of-plane magnetic field angle β is defined as shown in Supplementary Fig. 4a. $\tilde{V}_{\rm SSE}(\beta)$ is well fitted by a $\sin(\beta)$ function, which is consistent with the angular dependence of the inverse spin-Hall effect (ISHE) voltage: $V_{\rm ISHE} \propto [\mathbf{J}_{\rm s} \times \boldsymbol{\sigma}]_a \propto \sin(\beta)$. Here, $\mathbf{J}_{\rm s}$ and $\boldsymbol{\sigma}$ denote the spatial direction of the spin current and the spin-polarization vector of the spin current, respectively[6]. $[\mathbf{J}_{\rm s} \times \boldsymbol{\sigma}]_a$ is the a-axis component of $\mathbf{J}_{\rm s} \times \boldsymbol{\sigma}$.

The heater-power dependence of $V_{\rm SSE}$ is shown in Supplementary Fig. 4b. The amplitude

of V_{SSE} at each heater-power is estimated by fitting $V_{\mathrm{SSE}}(\beta)$ using a $\sin(\beta)$ curve at $\mu_0 H = 3$ T. When the current applied to the Au heater is less than 2 mA, the heater-power dependence of V_{SSE} is well fitted by a linear function. For higher heater power, however, V_{SSE} deviates from the linear dependence. This non-linear dependence was also observed in the magnon spin-Seebeck effect in $Y_3Fe_5O_{12}$/Pt systems[7]; the non-linearity is attributed to the strong non-equilibrium excitation of magnons in $Y_3Fe_5O_{12}$. Another possibility for the non-linearity in the $CuGeO_3$ case is temperature rise of the $CuGeO_3$ sample due to the Joule heating, since V_{SSE} should decrease with increasing T. Therefore, to avoid the undesirable non-equilibrium/heating effects, we fixed the heater current at 1.5 mA for all the measurements shown in the main text.

Supplementary Note E. Temperature and field dependence of the Pt and Au resistivity

V_{SSE} is proportional to the heating power of the Au heater. In the case of a constant current (I_{Au}), V_{SSE} is proportional to the resistance of Au (R_{Au}). V_{SSE} is also proportional to the resistance of Pt (R_{Pt}) for a constant spin current J_s injection: $V_{\mathrm{SSE}} \propto J_s \times R_{\mathrm{Pt}}$. Thus, a change in R_{Pt} and R_{Au} with respect to T and H will cause extrinsic variations in V_{SSE}. Supplementary Figs. 5a and b show the T dependence of R of the Pt wire and Au heater, respectively. As T is reduced, both R_{Pt} and R_{Au} decrease monotonically. As shown in the insets of Supplementary Figs. 5a and b, the variation of R_{Pt} and R_{Au} at low T ($T < 20$ K) is less than 1 %. The H-dependence of R_{Pt} and R_{Au} at $T = 2$ K is shown in Supplementary Figs. 5c and S5d. R_{Pt} and R_{Au} change less than 1 % up to 6 T in both metals. According to the $R(T)$ and $R(H)$ results, the contribution of the resistance change to the observed SSE signal is negligibly small.

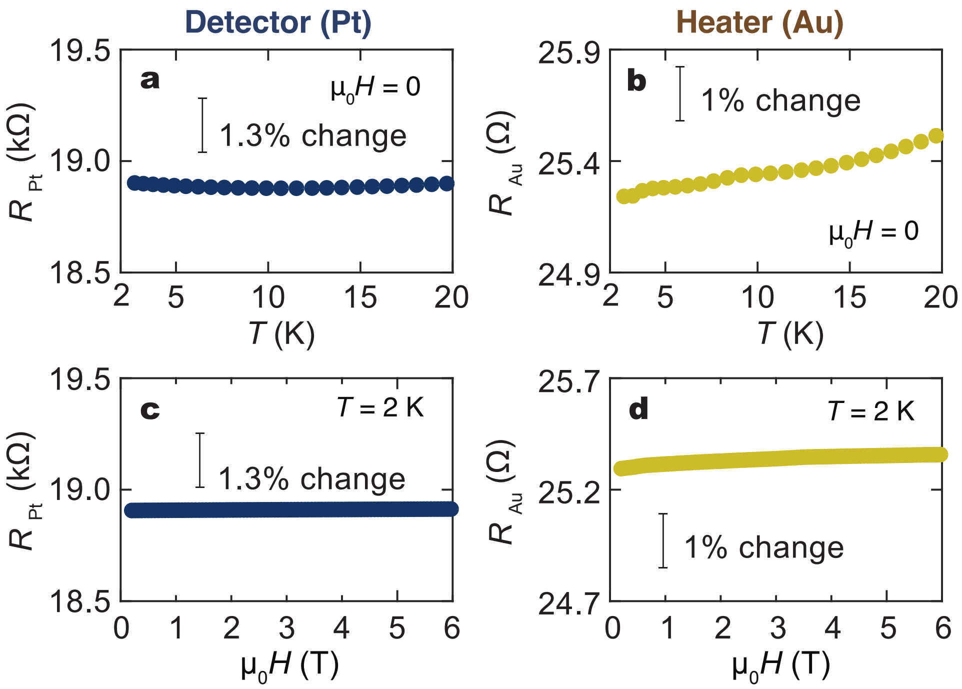

Supplementary Fig.5. **Temperature (T) and magnetic field (H) dependence of resistance (R) of detector and heater. a-b,** T dependence of R of Pt and Au. **c-d,** The magnetoresistance of Pt and Au at 2 K. The resistivity change for both metals up to 6 T is below 1 %.

As for the actual temperature at the interface, we can use the Pt detector layer as a thermometer (i.e. Pt resistance thermometer) to investigate the temperature change with and without the Au heating current (e.g. ref.[8]). When the heater current is applied to the Au heater, the resistance of the Pt detector changes, as shown in Supplementary Fig. 6a. Using dR/dT and $\Delta R \equiv R(I_{\rm Au} = 1.5{\rm mA}) - R(I_{\rm Au} = 0)$, we estimated the typical temperature difference across the device $\Delta T = \Delta R/({\rm d}R/{\rm d}T)$ to be 0.2-0.5 K, as shown in Supplementary Fig. 6d. For $T > 5$ K, ΔT is as small as 0.25 K and 0.5 K even at $T = 2$ K. In the constant power setup, a lower value of

thermal conductivity κ leads to a larger temperature difference and causes a larger observed SSE signal. In the case of $CuGeO_3$, because of the suppression of phonon transport, κ monotonically decreases for $T < 5$ K (ref. [9]), which is consistent with our results.

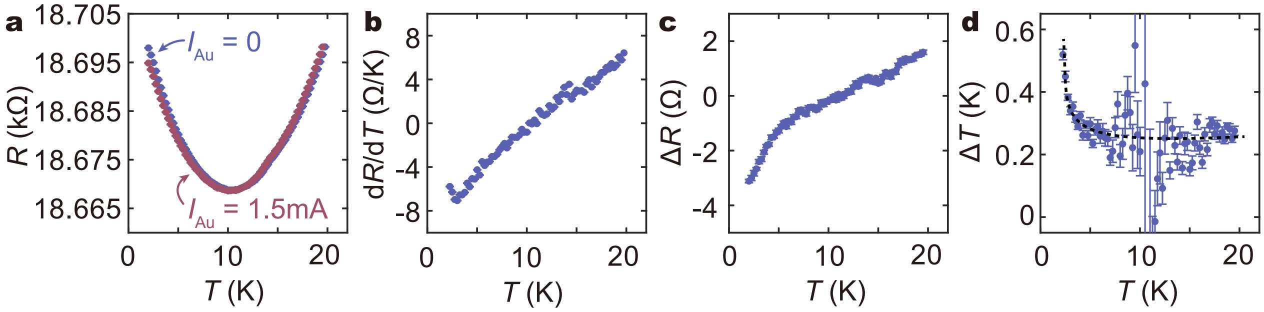

Supplementary Fig.6. **Temperature difference at the interface. a,** The temperature (T) dependence of the resistance (R) of Pt with and without the heating current (I_{Au}). **b-d,** The temperature dependence of $\mathrm{d}R/\mathrm{d}T$, ΔR and ΔT. The dotted curve is a guide for the eyes. Error bars represent standard deviation.

Supplementary Note F. Impurity density in $Cu_{0.99}Zn_{0.01}GeO_3$ and nominally pure $CuGeO_3$

The density of impurities in $CuGeO_3$ can roughly be estimated from the $\chi(T)$ curve. In the SP phase, the magnetic susceptibility can be decomposed into three terms[10,11]:

$$\chi(T) = \chi_0 + \chi_{\mathrm{Para}}(T) + \chi_{\mathrm{SP}}(T) \tag{1}$$

where χ_0, χ_{Para}, and χ_{SP} stand for constant diamagnetic contribution, paramagnetic contribution from impurity-induced free spins (unpaired Cu spins) obeying $\chi_{\mathrm{Para}} = C/(T - \Theta)$, and spin-Peierls contribution, respectively. For $Cu_{0.99}Zn_{0.01}GeO_3$ sample, $C = 5.1 \times 10^{-7}\ \mu_{\mathrm{B}}\mathrm{Cu} \cdot \mathrm{Oe}^{-1}$

were obtained by fitting the $\chi(T)$ data by using Supplementary Eq. (1) at a low T range (< 3 K), assuming that the spin-Peierls contribution is zero ($\chi_{\rm SP} = 0$)[11]. The fitted result of $\chi_{\rm Para}$ is shown as a function of T in Supplementary Fig. 7a. Assuming that all $S = 1/2$ Cu^{2+} ions contribute to the paramagnetic term, $C_{\rm all} = \frac{S(S+1)g^2\mu_B^2}{3k_B} = 7.06 \times 10^{-5}\mu_{\rm B}{\rm Cu}\cdot{\rm Oe}^{-1}$ with $g = 2.1$ for $CuGeO_3$ (ref. [12]). By comparing $C_{\rm all}$ with the fitted C result, we conclude that the density of free spins is about $C/C_{\rm all} \sim 0.73\%$ of all Cu atoms, which is very close to the 1% Zn-doping. Preforming the same analysis on the $CuGeO_3$ sample (see Supplementary Fig. 7b), we obtain an impurity density level ($C/C_{\rm all}$) of 0.02% in the $CuGeO_3$ sample. Therefore, even in nominally pure samples, the effect of triplon scattering by impurities cannot be ignored.

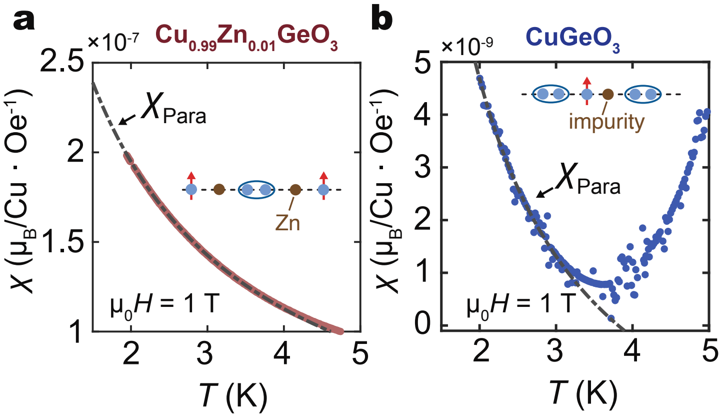

Supplementary Fig.7. **Magnetic susceptibility (χ) at the low temperature (T) range. a** and **b,** temperature (T) dependence of magnetic susceptibility (χ) for $Cu_{0.99}Zn_{0.01}GeO_3$ and $CuGeO_3$ at a low T range. The impurity induced paramagnetic term $\chi_{\rm Para}$ for each sample is estimated from the fitting to the Curie-Weiss law (black curves).

Supplementary Note G. Theoretical analysis of SSE in $CuGeO_3$

In this section, we discuss our theoretical analysis for SSE in $CuGeO_3$. First, we review a quantum sine-Gordon (SG) model as low-energy effective theory for $CuGeO_3$. The SG model describes triplon excitation in the spin-Peierls phase. Then, based on the results of the SG model, we compute a SSE spin current in $CuGeO_3$. To this end, we apply two microscopic methods: the Boltzmann equation and the tunnel spin-current theory based on Keldysh Green's function.

Supplementary Note G1. Quantum sine-Gordon field theory as the low-energy effective model for $CuGeO_3$

Here, we explain low-energy effective theory for $CuGeO_3$. The model Hamiltonian for each antiferromagnetic spin chain in $CuGeO_3$ is

$$H_{\rm SP} = \sum_j J_1(1+\delta(-1)^j)\mathbf{S}_j \cdot \mathbf{S}_{j+1} + J_2 \mathbf{S}_j \cdot \mathbf{S}_{j+2} - B\sum_j S_j^z. \quad (2)$$

where $\mathbf{S}_j$ is the electron spin on the j-th site, and $B \equiv g\mu_B\mu_0 H$ with the g factor $g = 2.1$ (ref. [12]), Bohr magneton μ_B and an external magnetic flux density $\mu_0 H$. For $CuGeO_3$, the exchange coupling constants of nearest neighboring spins and next nearest neighboring spins are respectively estimated to be $J_1 \simeq 120.6$ (ref. K[13]) and $J_2 \simeq 0.36 J_1$ (ref. [14]). A finite dimerization parameter, δ, emerges in the spin-Peierls phase due to the lattice deformation. First, we focus on the zero-field case. In the small-J_2 case of $\alpha \equiv J_2/J_1 < 0.241 \equiv \alpha_c$ (ref. [15]), the spin chain belongs to the universality class of a Tomonaga-Luttinger (TL) liquid, i.e., the ground state is a non-magnetic spin liquid and the low-energy excitations are described by gapless spinons. For $\alpha > \alpha_c$, the ground state is spontaneously dimerized and an excitation gap opens. A dimerization induces

a lattice distortion through the spin-phonon coupling, and, as a result, δ becomes finite. In the dimerized (i.e., spin-Peierls) phase with a finite δ, the lowest excitation branch is given by the triply degenerated $S = 1$ mode, namely, triplons. The low-energy effective Hamiltonian [16–18] is given by

$$H_{\rm eff} = \int dx \;\; \frac{v}{2\pi}\left[\frac{1}{K}(\partial_x\phi)^2 + K(\partial_x\theta)^2\right] + \frac{J_1\delta d}{a_0}\sin(\sqrt{4\pi}\phi) + \frac{\lambda}{a_0}\cos(\sqrt{16\pi}\phi). \tag{3}$$

Where ϕ and θ are a pair of dual boson fields. v and K are, respectively, the spinon velocity and the TL-liquid parameter, and a_0 is the lattice constant. The spin-rotation SU(2) symmetry makes the TL-liquid parameter fixed to be $K = 1/2$. The quadratic-boson (i.e., free-boson) part corresponds to the TL-liquid Hamiltonian. The sine term stems from the bond alternation δ and the non-universal constant $d(> 0)$ can be numerically determined [17,19] while the cosine term $\cos(\sqrt{16\pi}\phi)$ exists even in the uniform spin chain without δ. The sine term with scaling dimension K is more relevant than the cosine term with dimension $4K$. If α is close enough to α_c, the coupling constant $\lambda \propto \alpha - \alpha_c$. In the uniform case of $\delta = 0$, the λ term is marginally irrelevant (relevant) for $\lambda < 0$ ($\lambda > 0$), which corresponds to a gapless TL liquid (a spontaneously dimerized ground state with a spin gap). For a SP phase with $\delta \neq 0$, a small λ term can be negligible in the sense of renormalization group, and the low-energy Hamiltonian is given by a sum of the TL-liquid part and a single cosine term $\cos(\sqrt{4\pi}\phi)$. This is called quantum sine-Gordon (SG) model and is an integrable quantum field theory [16,18,20,21]. Since α is close to α_c in $CuGeO_3$, we can expect that its low-energy physics is described by a SG model. The SG model is known to well describe the low-energy physics of the spin-Peierls Hamiltonian with high accuracy [16–19], and the model enables us to compute various physical quantities in a quantitative level [22,23].

The sine term is relevant in the SU(2) case we focus on, and therefore the field ϕ is locked at the energy minimum of the sine potential in the ground state, i.e., $\langle\phi\rangle = \sqrt{\pi}(n - \frac{1}{4})$ for $\delta > 0$ and $\langle\phi\rangle = \sqrt{\pi}(n + \frac{1}{4})$ for $\delta < 0$ (n: integer). The elementary excitations in the SG model are soliton, antisoliton, and breathers (bound state of soliton and antisoliton) [17,20,21]. The soliton and antisoliton are the domain-wall excitations connecting two neighboring ground states: For instance, a soliton texture located around $x = x_0$ is given by $\langle\phi\rangle = \sqrt{\pi}(n - \frac{1}{4})$ for $x \ll x_0$ and $\langle\phi\rangle = \sqrt{\pi}(n + 1 - \frac{1}{4})$ for $x \gg x_0$. The mass gap of these excitations depends on the parameters v, K, and $J_1\delta d/a_0$ if we neglect the marginal cosine term $\cos(\sqrt{16\pi}\phi)$. In the SU(2) case, soliton, antisoliton, and first breather have the same mass $m_1 \sim \delta^{2/3}$. Second breather's mass m_2 is given by $m_2 = \sqrt{2}m_1$. In the language of spin-Peierls chains, the triply-degenerated excitations of soliton, antisoliton, and first breather correspond to spin-1 triplons around the wave number $k = \pi$, while the second breather is spin-singlet excitation around $k = \pi$. The energy bands of triplons, i.e., soliton ($S_z = 1$), first breather ($S_z = 0$), and anti-soliton ($S_z = -1$), are respectively given by

$$\epsilon_{\rm S}(k_\pi) = \sqrt{m_1^2 + v^2k_\pi^2} - B, \qquad \text{for } S_z = 1; \tag{4}$$

$$\epsilon_{\rm B}(k_\pi) = \sqrt{m_1^2 + v^2k_\pi^2}, \qquad \text{for } S_z = 0; \tag{5}$$

$$\epsilon_{\rm AS}(k_\pi) = \sqrt{m_1^2 + v^2k_\pi^2} + B, \qquad \text{for } S_z = -1. \tag{6}$$

where $k_\pi = k - \pi$. The B dependence of $\epsilon_{\rm AS}$ and $\epsilon_{\rm S}$ reflects the Zeeman splitting.

Finally, we mention values of the parameters in the SG model for $CuGeO_3$. We have $K = 1/2$ in the SU(2) model, and the spin gap (triplon gap) $m_1 \simeq 2.36$ meV (≈ 27.4 K) is estimated from an ESR experiment[12] at a low temperature. The velocity v may be approximated by that of J_1-

J_2^c spin chains since α of $CuGeO_3$ is closed to α_c. The velocity has been numerically estimated as $v = 1.174 J_1 a_0$ (refs. [17,24]). Combining this value of v and the exact result of the SG model[17,20], we can compute the soliton mass as $m_1/J_1 \simeq 2.13\delta^{2/3}$. This relation, $J_1 \simeq 120.6$ K, and $m_1 \simeq 27.4$ K lead to $\delta \simeq 0.034$ in $CuGeO_3$ at a sufficiently low temperature. If temperature T grows up to the same order as the lowest gap $\epsilon_S(0) = m_1 - B$, the fluctuation of δ increases mainly due to the effect of phonons, and as a result, the description of the SG model gradually becomes invalid.

Supplementary Note G2. Approach based on Boltzmann equation

Using the above result, let us compute the thermal spin current in SSE for $CuGeO_3$. The SG model predicts that when magnetic field B is increased in the SP phase, the difference of the soliton density and anti-soliton one (i.e., spin current carrier) monotonically increases at a fixed temperature due to the Zeeman splitting. Nevertheless, the experimental result shows a non-monotonic B dependence of the SSE voltage. It indicates the importance of scattering processes among triplons, impurities, phonons, etc.

If we consider a triplon as a wave packet located at $\boldsymbol{r}$ with the wave vector $\mathbf{k}$, the triplon distribution function $f_\eta(\mathbf{k}, \mathbf{r}, t)$ follows the Boltzmann equation,

$$\frac{\partial}{\partial t} f_\eta + \mathbf{v}_\eta(\mathbf{k}) \cdot \nabla_{\mathbf{r}} f_\eta + \frac{d\mathbf{k}}{dt} \cdot \nabla_{\mathbf{k}} f_\eta = \frac{\partial}{\partial t} f_\eta|_{\text{scattering}}, \tag{7}$$

where the index η corresponds to soliton ($S_z = -1$) and anti-soliton ($S_z = 1$). The right hand side is the scattering rate of triplons. The group velocity of trilpon is $\mathbf{v}_\eta(\mathbf{k}) = \frac{1}{\hbar}\frac{\partial \epsilon_\eta(\mathbf{k})}{\partial \mathbf{k}}$. Since breathers are spin-singlet quasi particles and do not contribute to spin current. Therefore, it is enough to consider soliton and anti-soliton for computing the spin current.

The real compound $CuGeO_3$ has a weak but finite three dimensionality, but we expect that the triplon distribution along the spin-chain (x) direction is essential for the SSE in $CuGeO_3$. Therefore, we will replace $\mathbf{k}$ with the wave number k along the x direction hereafter. In the SSE measurement, a temperature gradient is applied along the x axis to the sample. If the system length L_x is sufficiently large, we may approximate the gradient as the constant $\partial T/\partial x = -\frac{T_{\rm high}-T_{\rm low}}{L_x} \equiv \Delta T_x$. For a small ΔT_x, the system approaches to a non-equilibrium steady state following local equilibrium, and the distribution function is approximated as

$$f_\eta = f_\eta^{(0)}(k) + g_\eta(k,x,t), \tag{8}$$

where $f_\eta^{(0)}(k)$ is the equilibrium distribution function and $g_\eta(k,x,t)$ is the deviation from the equilibrium. We assume that $f_\eta^{(0)}$ can be described by a Bose distribution function,

$$f_\eta^{(0)}(k) = \frac{1}{e^{\beta(\epsilon_\eta(k)-\mu)} - 1}. \tag{9}$$

Triplons are not equal to bosons or fermions, but the approximation of Supplementary Eq. (9) is expected to work well if triplon density is low enough. The non-equilibrium steady state satisfies $\partial f_\eta/\partial t = dk/dt = 0$. For the scattering term, we adapt relaxation time (τ_k) approximation [25]:

$$\frac{\partial}{\partial t} f_\eta|_{\rm scattering} = -\frac{f_\eta(k,x,t) - f_\eta^{(0)}(k)}{\tau_k} = -\frac{g_\eta(k,x,t)}{\tau_k}. \tag{10}$$

Under the assumption that $\partial T/\partial x$ and g are small, we may omit the $\partial g/\partial T$ term in Supplementary Eq. (7) and therefore it is simplified as

$$g_\eta(k) = \tau_k v_\eta(k) k_B \Delta_x T \frac{\partial}{\partial(k_B T)} f_\eta^{(0)}(k) \tag{11}$$

From these discussions, the total spin current with S^z polarization along the x direction is

$$\begin{aligned}
J_s &= \sum_{S_z=1,-1}\sum_k \hbar S_z v_{S_z}(k) f_{S_z}(k) \\
&= \sum_{S_z}\sum_k \hbar S_z v_{S_z}(k) g_{S_z}(k) \\
&= \sum_{S_z}\sum_k \hbar S_z v_{S_z}(k)^2 \tau_k k_B \Delta_x T \frac{\epsilon_{S_z}(k)}{(k_B T)^2}\frac{e^{\beta\epsilon_{S_z}(k)}}{(e^{\beta\epsilon_{S_z}(k)}-1)^2} \\
&= \sum_{S_z}\int_{\mathrm{B.Z.}} \frac{dk}{2\pi}\hbar S_z v_{S_z}(k)^2 \tau_k k_B \Delta_x T \frac{\epsilon_{S_z}(k)}{(k_B T)^2}\frac{e^{\beta\epsilon_{S_z}(k)}}{(e^{\beta\epsilon_{S_z}(k)}-1)^2}.
\end{aligned} \tag{12}$$

This is Equation (1) in the main text.

To numerically calculate the H dependence of J_s, it is necessary to determine the relaxation time τ_k based on appropriate impurity scattering mechanisms. If we focus on the elastic scattering between a triplon and an impurity, τ_k is estimated as [25]

$$\tau_k(\epsilon)^{-1} \sim n_{\mathrm{imp}} D(\epsilon) V^2, \tag{13}$$

where n_{imp}, $D(\epsilon)$ and V are impurity density, the density of states (DOS) of the triplons and impurity potential, respectively. In $CuGeO_3$, $J_c \sim 120$, $J_b \sim 0.1 J_c$ and $J_a \sim -0.01 J_c$ (ref. [13]), and the two-dimensionality is strong compare to other one-dimensional systems. Therefore, We may approximate $D(\epsilon)$ at the bottom of the band as a constant. Consider the elastic scattering from both nonmagnetic and magnetic impurities, the total relaxation time can be approximated as

$$\tau_{k,\mathrm{total}}^{-1} = \tau_{k,\mathrm{non-mag}}^{-1} + \tau_{k,\mathrm{mag}}^{-1}, \tag{14}$$

if the impurity densities are small enough. Combining these theoretical arguments and experimental results on the doped $CuGeO_3$, we assume that the relaxation times from nonmagnetic and

magnetic impurities is given by

$$\tau_{k,\mathrm{non-mag}}^{-1} = n_{\mathrm{imp}} C_0 V_0^2; \tag{15}$$

$$\tau_{k,\mathrm{mag}}^{-1} = \Big(n_{\mathrm{imp}} B_S(T,H)\Big) \cdot C_{\mathrm{mag}} \cdot \Big(V_{\mathrm{mag}} B_S(T,H)\Big)^2. \tag{16}$$

C_0 and C_{mag} are constants and $B_S(T,H)$ is the Brillouin function with $S = 1/2$. The introduction of B_S is reasonable because it is expected that magnetically-polarized impurities more strongly refute triplons compared to weakly polarized ones. The numerical result of $J_s(H)$ at $T = 2.4$ K is shown in the main text as Fig. 4, in which $C_{\mathrm{mag}} V_{\mathrm{mag}} \gg C_0 V_0$ and the T dependent triplon gap $m_1(T)$ measured in the neutron scattering are used. Due to the Zeeman-splitting induced density difference between soliton and antisoliton, the sign of spin current is opposite to that in ferromagnetic insulators. We verify that the broad peak structure of J_s as a function of H is stable against small changes of $\tau_{k,\mathrm{mag}}^{-1}$. Moreover, the broad peak still survives even if the equilibrium distribution $f_\eta^{(0)}$ is changed into the fermion one in the regime of a low triplon density.

Supplementary Note G3. Approach based on the tunnel spin current

Another microscopic approach for SSE is the formula of tunnel spin current on the interface from the magnet ($CuGeO_3$) to the metal (Pt)[2,26–28]. An advantage of this approach is that it can be applied to a broad class of magnets even with non-magnon type excitations such as spinons, triplons, magnon pairs, etc. However, it cannot take into account the effects of scattering among magnetic excitations during a flow of spin currents.

In this approach, we focus on the interface between $CuGeO_3$ and Pt. We assume that the dominant interaction at the interface is Heisenberg type exchange interaction between localized

spin of $CuGeO_3$ and conducting-electron spin of Pt. Such an exchange interaction has been used to describe interfacial spin transfer between an insulator magnet and a metal[2,26–28]. The interface Hamiltonian is given by

$$H_{\rm int} = J_{\rm int} \sum_{\mathbf{r}\in{\rm interface}} \mathbf{S}_{\mathbf{r}} \cdot \mathbf{s}_{\mathbf{r}}, \tag{17}$$

where $\mathbf{S}_{\mathbf{r}}$ and $\mathbf{s}_{\mathbf{r}}$ respectively denote the localized spin and the conducting-electron spin at site $\mathbf{r}$ on the interaface. If we perturbatively treat this interface interaction using Keldysh Green's function method [27,29], the tunneling spin current is calculated as

$$I_s \propto -J_{\rm int}^2 \int_{-\infty}^{\infty} d\omega {\rm Im} X_R^{-+}(\omega) {\rm Im}\chi_R^{-+}(\omega) \left[\coth\left(\frac{\omega}{2k_B T_{\rm metal}}\right) - \coth\left(\frac{\omega}{2k_B T_{\rm magnet}}\right)\right] \tag{18}$$

up to the leading order of the interface coupling constant $J_{\rm int}$. Here, ω, $T_{\rm metal}$ and $T_{\rm magnet}$ are respectively frequency, the spatially averaged value of temperature in the metal (Pt), and that in the $CuGeO_3$. $X_R^{-+}(\omega)$ and $\chi_R^{-+}(\omega)$ are respectively the retarded part of the local dynamical susceptibility of the $CuGeO_3$ at $T = T_{\rm magnet}$ and that of the Pt at $T = T_{\rm metal}$. The indices $^{-+}$ denotes the transverse spins $S^{\pm}$. $-{\rm Im}X_R^{-+}(\omega)$ with $\omega > 0$ may be viewed as the DOS of $S_z = +1$ magnetic excitations, while ${\rm Im}X_R^{-+}(\omega)$ with $\omega < 0$ as that of $S_z = -1$ magnetic excitations[2]. On the other hand, the magnetic energy scale in $CuGeO_3$ is much smaller than that of the kinetic energy of electrons in the metal (Pt), and therefore the susceptibility of the metal can be approximated as[30,31]

$${\rm Im}\chi_R^{-+}(\omega) \simeq \hbar\omega D(\epsilon_F)^2 + \cdots, \tag{19}$$

where $D(\epsilon_F)$ is the DOS of conduction electrons at Fermi surface $\epsilon = \epsilon_F$ in the metal. Namely, ${\rm Im}\chi_R^{-+}(\omega)$ is an odd function of ω. Therefore, the sign of the tunnel spin current reflects the difference between the weights of $S_z = -1$ and $S_z = +1$ modes. If the temperature difference

$\Delta T = (T_{\rm magnet} - T_{\rm metal})/2$ is sufficiently small, the T-dependent factor in Supplementary Eq. (18) is approximated by

$$\coth\left(\frac{\omega}{2k_BT_{\rm metal}}\right) - \coth\left(\frac{\omega}{2k_BT_{\rm magnet}}\right) \simeq -\frac{\omega}{(k_BT_{\rm ave})^2}\frac{1}{\sinh^2\left(\frac{\omega}{2k_BT_{\rm ave}}\right)}\Delta T + \cdots , \quad (20)$$

where $T_{\rm ave}$ is the averaged temperature $(T_{\rm magnet} + T_{\rm metal})/2$. Substituting Supplementary Eq. (19) and (20) into Supplementary Eq. (18), we arrive at the simplified formula

$$I_s \propto \pi J_{\rm int}^2 D(\epsilon_F)^2 \Delta T \frac{1}{(k_BT)^2}\int_{-\infty}^{\infty} d\omega {\rm Im} X_R^{-+}(\omega)\frac{\omega^2}{\sinh^2\left(\frac{\omega}{2k_BT}\right)}, \quad (21)$$

where for simplicity we have replaced $T_{\rm ave}$ with T.

Here, we emphasise that the formula of this tunnel spin current has suceeded in well explaining several SSEs in standard ferro(i)magnets[26], an one-dimensional spin liquid[2], a spin-nematic magnet[28], and a compensated ferrimagnet[32]. We also note two points about the formula of Supplementary Eq. (18). The first thing is that triplons in the magnetic insulator $CuGeO_3$ cannot be injected to the metal Pt, while the spin of triplons is transfered to that of conducting electrons in Pt through the interface exchange $J_{\rm int}$. Namely, spin angular momentum can tunnel from $CuGeO_3$ to Pt and vice versa. This is in contrast with a tunnel charge current in bilayer systems consisiting of two metals. The second is about the possibility of the T and H dependences of $J_{\rm int}$. The energy scale of chemical bonds at the interface is usually higher than those of the ranges of T and H (0 - 15 K and 0 - 14 T). Therefore, we have here assumed that $J_{\rm int}$ is independent of T and H.

The remaining task is to compute the susceptibility for a spin-Peierls chain ${\rm Im}X_R^{-+}(\omega)$. If $CuGeO_3$ in the spin-Peierls phase is described by the SG model, we can use so-called the form-

factor method [21]. Following it, we calculate the dynamical susceptibility in (k, ω) space as

$$X_R^{-+}(k_\pi, \omega) \simeq \frac{-Z}{\omega + \epsilon_{\rm AS}(k_\pi) + i\gamma} + \frac{Z}{\omega - \epsilon_{\rm S}(k_\pi) + i\gamma} + \cdots , \tag{22}$$

around $k = \pi$. Here the first and second terms are respectively the contribution of antisoliton ($S_z = -1$) and soliton ($S_z = 1$), and $\gamma \to +0$ is the infinitesimal factor. The renormalization factor Z generally depends on k and ω, but it can be viewed as a constant if the system is ideally described by the integrable SG model. The local susceptibility $X_R^{-+}(\omega)$ is related to $X_R^{-+}(k_\pi, \omega)$ as $X_R^{-+}(\omega) = N^{-1} \sum_{k_\pi} X_R^{-+}(k_\pi, \omega)$ with N being the total site number. As we mentioned, in finite temperature case, the neutron-scattering experiments showed that the mass gaps of soliton and antisoliton decrease and their life time becomes shorter. This behavior cannot be reproduced within the SG model, but such T dependence can be treated by replacing γ and m_1 of the bands $\epsilon_{\rm S,AS}$ with the line width $\Gamma(T)$ and the T-dependent mass $m_1(T)$, respectively. Namely, we may describe the finite-temperature susceptibility $X_R^{-+}(k_\pi, \omega)$ as

$$X_R^{-+}(k_\pi, \omega) \simeq \frac{-Z}{\omega + \epsilon_{\rm AS}(k_\pi, T) + i\Gamma(T)} + \frac{Z}{\omega - \epsilon_{\rm S}(k_\pi, T) + i\Gamma(T)} + \cdots , \tag{23}$$

where the bands $\epsilon_{\rm S,AS}(k_\pi, T)$ are defined as $\epsilon_{\rm S}(k_\pi, T) = \sqrt{m_1(T)^2 + v^2 k_\pi^2} - B$ and $\epsilon_{\rm AS}(k_\pi, T) = \sqrt{m_1(T)^2 + v^2 k_\pi^2} + B$.

Using Supplementary Eq. (21) and Supplementary Eq. (23), we can calculate the H and T dependence of the tunnel spin current in a low-T and low-H regime. The calculated result shows that a spin current linearly emerges as the field B increases and its sign is opposite to that of SSE in ferromagnetic insulators. These results in the small-B regime are consistent with those from the Boltzmann equation.

Supplementary Note G4. SSE in a moderate temperature regime

To compute the T-dependence of J_s in a relatively high temperature range (5 K - 10 K), we have to include the T-dependence of triplon bands and scattering processes in the Boltzmann equation approach. To this end, we need information on the T-dependence of the excitation gap of triplons $m_1(T)$ and the lifetime of triplons $1/\tau_k(T)$. By fitting neutron scattering results of spin-gap $m_1(T)$ (ref. [33]) with $m_1(T) = m_1(0)\big(1 - T/T_{\rm SP}\big)^{\alpha}$, we obtain $m_1(0) \sim 2.05$ meV and $\alpha \simeq 0.12$. In the numerical calculations, m_1 in Supplementary Eq. (4)-(6) is replaced by the fitted function of $m_1(T)$. The T-dependence of the lifetime of triplons is obtained as the peak width $\Gamma(T)$ of triplon excitation from inelastic neutron scattering experiments[33,34]. Here, the explicit T-dependence of $1/\tau_k$ is set as a new term $1/\tau_k(T)$, and the total relaxation time is

$$\tau_{k,\rm total}^{-1} = \tau_{k,\rm non-mag}^{-1} + \tau_{k,\rm mag}^{-1} + \tau_k(T)^{-1}. \tag{24}$$

$1/\tau_{k,\rm non-mag}$ is independent of T and $1/\tau_{k,\rm mag}$ only weakly depends on T via the Brillouin function.

The experimental results of triplon peak width $\Gamma(T)$ obtained from Ref. [34,35] are well fitted by a power law $\Gamma(T) = \Gamma(0)T^4$, where $\Gamma(0)$ is the fitting parameter. The scattering with thermally excited phonons and other triplons may be two origins of the T-dependence of Γ but the detail of microscopic mechanism is not clear. In this work, we treat these T-dependent scattering processes phenomenologically. Specifically, we set $\tau_k(T)$ as

$$\tau_k(T)^{-1} = C_{\rm th} V_{\rm th}^2 \Gamma(0) \Big(\frac{T}{T_{\rm SP}}\Big)^4. \tag{25}$$

Where $C_{\rm th}$ and $V_{\rm th}$ are T-independent constants.

Calculated result of the T-dependent SSE is shown in Supplementary Fig. 8. We have a

qualitative agreement between experiment and theory in the moderate temperature range (approximately 5 K - 10 K).

Finally, we emphasize that the result of the temperature dependence is justified only in the moderate temperature range. The reason why our result is less reliable in the high-T range ($T \to T_{\rm SP}$) is as follows: our theory is based on the Sine-Gordon model, which can well describe the low-energy physics of the SP phase. When T or H increases, the dimerized ground state is partially broken down and the SG model gradually becomes less reliable. For $T \to T_{\rm SP}$, the critical nature of the SP transition becomes relevant, and the triplon picture is no longer applicable. This is also why theoretical calculations in Supplementary Notes G1-G3 focused only on the low temperatures and low magnetic fields, in which the singlet ground state and triplon excitation are well established. On the other hand, the relation of $\Gamma(T) \propto T^4$ is not experimentally supported in the low-T range. Thus, Supplementary Fig. 8 shows the temperature range from 5 K to 11 K.

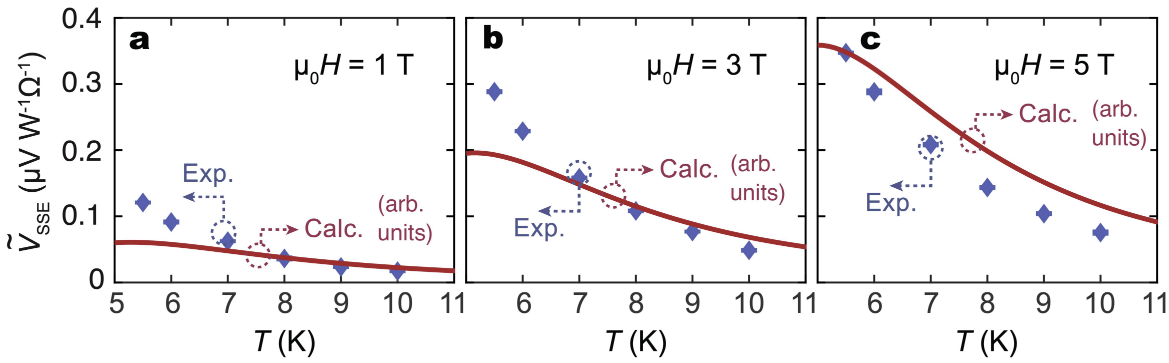

Supplementary Fig.8. **Comparison with theory. a-c** Comparison between calculation results of the T dependence of the triplon spin current and the observed SSE signal at $\mu_0 H = 1, 3, 5$ T, respectively. Error bars represent standard deviation.

1. Fujita, M. *et al.* Temperature Dependence of Spin Excitations in the Frustrated Spin Chain System CuGeO 3. *Journal of the Physical Society of Japan* **82**, 084708 (2013).

2. Hirobe, D. *et al.* One-dimensional spinon spin currents. *Nature Physics* **13**, 30–34 (2017).

3. Giamarchi, T., Rüegg, C. & Tchernyshyov, O. Bose-Einstein condensation in magnetic insulators. *Nature Physics* **4**, 198–204 (2008).

4. Seki, S. *et al.* Thermal Generation of Spin Current in an Antiferromagnet. *Phys. Rev. Lett.* **115**, 266601 (2015).

5. Wu, S. M. *et al.* Antiferromagnetic Spin Seebeck Effect. *Phys. Rev. Lett.* **116**, 097204 (2016).

6. Maekawa, S., Valenzuela, S., Saitoh, E. & Kimura, T. *Spin Current* (OUP Oxford, Oxford, 2012).

7. Oyanagi, K., Kikkawa, T. & Saitoh, E. Magnetic field dependence of the nonlocal spin Seebeck effect in Pt/YIG/Pt systems at low temperatures. *AIP Advances* **10**, 015031 (2020).

8. Wu, S. M., Fradin, F. Y., Hoffman, J., Hoffmann, A. & Bhattacharya, A. Spin Seebeck devices using local on-chip heating. *Journal of Applied Physics* **117**, 17C509 (2015).

9. Salce, B. *et al.* Thermal conductivity of pure and Si-doped $CuGeO_3$. *Physics Letters A* **245**, 1560–1563 (1998).

10. Hase, M., Terasaki, I. & Uchinokura, K. Observation of the spin-Peierls transition in linear Cu^{2+} (Spin-$\frac{1}{2}$) chians in an inorganic compound $CuGeO_3$. *Physical Review Letters* **70**, 3651–3654 (1993).

11. Grenier, B. *et al.* Magnetic susceptibility and phase diagram of $CuGe_{1-x}Si_xO_3$ single crystals. *Physical Review B* **57**, 3444–3453 (1998).

12. Nojiri, H. *et al.* Submillimeter Wave ESR Study of Spin Gap Excitations in $CuGeO_3$. *Journal of the Physical Society of Japan* **68**, 3417–3423 (1999).

13. Nishi, M., Fujita, O. & Akimitsu, J. Neutron-scattering study on the spin-Peierls transition in a quasi-one-dimensional magnet $CuGeO_3$. *Physical Review B* **50**, 6508–6510 (1994).

14. Kamieniarz, G., Bieliński, M. & Renard, J. P. Susceptibility behavior of $CuGeO_3$: Comparison between experiment and the quantum transfer-matrix approach. *Physical Review B* **60**, 14521–14524 (1999).

15. Okamoto, K. & Nomura, K. Fluid-dimer critical point in S = 1/2 antiferromagnetic Heisenberg chain with next nearest neighbor interactions. *Physics Letters A* **169**, 433–437 (1992).

16. Giamarchi, T. *Quantum Physics In One Dimension* (Clarendon Press, Oxford, 2003).

17. Takayoshi, S. & Sato, M. Coefficients of bosonized dimer operators in spin-$\frac{1}{2}$ XXZ chains and their applications. *Physical Review B* **82**, 214420 (2010).

18. Gogolin, A., Nersesyan, A. & Tsvelik, A. *Bosonization and Strongly Correlated Systems* (Cambridge University Press, 2004).

19. Hikihara, T., Furusaki, A. & Lukyanov, S. Dimer correlation amplitudes and dimer excitation gap in spin-1/2 XXZ and Heisenberg chains. *Physical Review B* **96**, 134429 (2017).

20. Lukyanov, S. & Zamolodchikov, A. Exact expectation values of local fields in the quantum sine-Gordon model. *Nuclear Physics B* **493**, 571–587 (1997).

21. Kuzmenko, I. & ESSLER, F. H. L. Dynamical correlations of the spin-1/2 Heisenberg XXZ chain in a staggered field. *Physical Review B* **79**, 024402 (2009).

22. Katsura, H., Sato, M., Furuta, T. & Nagaosa, N. Theory of the Optical Conductivity of Spin Liquid States in One-Dimensional Mott Insulators. *Physical Review Letters* **103**, 177402 (2009).

23. Sato, M., Katsura, H. & Nagaosa, N. Theory of Raman Scattering in One-Dimensional Quantum Spin-$\frac{1}{2}$ Antiferromagnets. *Physical Review Letters* **108**, 237401 (2012).

24. Okamoto, K. & Nakamura, T. Critical properties of the spin- Heisenberg chain with frustration and bond alternation. *Journal of Physics A: Mathematical and General* **30**, 6287–6298 (1997).

25. Abrikosov, A. *Fundamentals of the Theory of Metals* (Dover Publications, Mineola, New York, 2017).

26. Adachi, H., Ohe, J.-i., Takahashi, S. & Maekawa, S. Linear-response theory of spin Seebeck effect in ferromagnetic insulators. *Physical Review B* **83**, 533 (2011).

27. Jauho, A.-P., Wingreen, N. S. & Meir, Y. Time-dependent transport in interacting and noninteracting resonant-tunneling systems. *Physical Review B* **50**, 5528 (1994).

28. Hirobe, D. *et al.* Magnon Pairs and Spin-Nematic Correlation in the Spin Seebeck Effect. *Physical Review Letters* **123**, 117202 (2019).

29. Haug, H. & Jauho, A. *Quantum Kinetics In Transport And Optics Of Semiconductors* (Springer, Berlin, Heidelberg, 2007).

30. Moriya, T. The Effect of Electron-Electron Interaction on the Nuclear Spin Relaxation in Metals. *Journal of the Physical Society of Japan* **18**, 516–520 (1963).

31. Shastry, B. S. & Abrahams, E. What does the Korringa ratio measure? *Physical Review Letters* **72**, 1933–1936 (1994).

32. Geprägs, S. *et al.* Origin of the spin Seebeck effect in compensated ferrimagnets. *Nature Communications* **7**, 1–6 (2016).

33. Lussier, J. G., Coad, S. M., McMorrow, D. F. & Paul, D. M. The temperature dependence of the spin-Peierls energy gap in $CuGeO_3$. *Journal of Physics: Condens. Matter* **8**, L59–L64 (1996).

34. Regnault, L. P., Ain, M., Hennion, B., Dhalenne, G. & Revcolevschi, A. Inelastic-neutron-scattering investigation of the spin-Peierls system $CuGeO_3$. *Physical Review B* **53**, 5579–5597 (1996).

35. Kikuchi, J., Yasuoka, H., Hase, M., Sasago, Y. & Uchinokura, K. Cu nuclear quadrupole resonance study of cugeo3. *Journal of the Physical Society of Japan* **63**, 872–875 (1994).
